# Confirmation of a metastasis-specific microRNA signature in primary colon cancer

**DOI:** 10.1038/s41598-018-22532-1

**Published:** 2018-03-27

**Authors:** Robert R. J Coebergh van den Braak, Anieta M. Sieuwerts, Zarina S. Lalmahomed, Marcel Smid, Saskia M. Wilting, Sandra I. Bril, Shanshan Xiang, Michelle van der Vlugt-Daane, Vanja de Weerd, Anne van Galen, Katharina Biermann, J. Han J. M van Krieken, Wigard P. Kloosterman, John A. Foekens, MATCH study group*, Peter-Paul L. O. Coene, Jan Willem T. Dekker, David D. E. Zimmerman, Geert W. M. Tetteroo, Wouter J. Vles, Wietske W. Vrijland, John W. M. Martens, Jan N. M. IJzermans

**Affiliations:** 1 Department of Surgery, Erasmus MC Medical Center, ‘s Gravendijkwal 230, 3015 CE Rotterdam, The Netherlands; 2 Department of Medical Oncology, Erasmus MC Cancer Institute, Erasmus University Medical Center, Rotterdam, The Netherlands; 3 Cancer Genomics Center Netherlands, Amsterdam, The Netherlands; 4 Department of Pathology, Erasmus MC Medical Center, Rotterdam, The Netherlands; 5 Department of Pathology, Radboud UMC, Geert Grooteplein Zuid 10, 6525 GA Nijmegen, The Netherlands; 6 Department of Genetics, Center for Molecular Medicine, University Medical Center Utrecht, Heidelberglaan 100, 3584 CX Utrecht, The Netherlands; 7 Maasstad Ziekenhuis, Maasstadweg 21, 3079 DZ Rotterdam, The Netherlands; 8 Reinier de Graaf Gasthuis, Reinier de Graafweg 5, 2625 AD Delft, The Netherlands; 9 Elisabeth-Tweesteden Ziekenhuis, Hilvarenbeekseweg 60, 5022 GC Tilburg, The Netherlands; 10 IJsselland Ziekenhuis, Prins Constantijnweg 2, 2906 ZC Capelle aan den IJssel, The Netherlands; 11 Ikazia Ziekenhuis, Montessoriweg 1, 3083 AN Rotterdam, The Netherlands; 12 Franciscus Gasthuis & Vlietland, Kleiweg 500, 3045 PM Rotterdam, The Netherlands

## Abstract

The identification of patients with high-risk stage II colon cancer who may benefit from adjuvant therapy may allow the clinical approach to be tailored for these patients based on an understanding of tumour biology. MicroRNAs have been proposed as markers of the prognosis or treatment response in colorectal cancer. Recently, a 2-microRNA signature (*let-7i* and *miR-10b*) was proposed to identify colorectal cancer patients at risk of developing distant metastasis. We assessed the prognostic value of this signature and additional candidate microRNAs in an independent, clinically well-defined, prospectively collected cohort of primary colon cancer patients including stage I-II colon cancer without and stage III colon cancer with adjuvant treatment. The 2-microRNA signature specifically predicted hepatic recurrence in the stage I-II group, but not the overall ability to develop distant metastasis. The addition of *miR-30b* to the 2-microRNA signature allowed the prediction of both distant metastasis and hepatic recurrence in patients with stage I-II colon cancer who did not receive adjuvant chemotherapy. Available gene expression data allowed us to associate *miR-30b* expression with axon guidance and *let-7i* expression with cell adhesion, migration, and motility.

## Introduction

Colorectal cancer (CRC) is the second most common malignancy in the Western world, with nearly 450,000 new cases in Europe in 2012^1^. As in most solid cancers, histological tumour staging (TNM) is currently the best determinant for prognosis and treatment. The current treatment for colon cancer is surgery alone for stages I and II and surgery followed by adjuvant chemotherapy for stage III. Despite treatment, up to 21% of patients with stage I-II colon cancer and up to 40% of patients with stage III colon cancer will develop metastatic disease after curative surgery^2,3^. Therefore, prognostic biomarkers complementing the TNM classification are urgently needed^4,5^. The only biomarker currently used to predict the prognosis and response to therapy in resectable colon cancer is microsatellite instability (MSI), a phenotype associated with a favourable prognosis compared to microsatellite stable (MSS) tumours.^6^

MicroRNAs (miRNAs) are a group of short noncoding RNAs that regulate gene expression at the post-transcriptional level^7^. In cancer, miRNAs play a central role in key pathways. In CRC, a growing number of miRNAs have been connected to different steps of tumourigenesis and been proposed as markers of prognosis or the treatment response^8^. Recently, Hur *et al*. identified six miRNAs as potential markers of the development of metastases in CRC patients (*miR-320*, *miR-221*, *miR-30b*, *miR-10b*, *miR-885-5p*, and *let-7i*) via a metastasis-specific miRNA biomarker discovery approach showing differential expression of these six miRNAs between primary CRC and paired metastatic liver tissues^9^. Two of these miRNAs (*miR-10b* and *let-7i*) measured in primary tumours were associated with the development of distant metastases. The combined expression of these two miRNAs identified a group of patients that remained entirely free of distant metastases.

In this study, we aimed to assess the prognostic value of the above-mentioned miRNAs and the 2-miRNA metastasis-specific signature in an independent, clinically well-defined, prospectively collected cohort of primary colon cancers. Patients with lymph node negative (LNN) colon cancer who did not receive systemic adjuvant chemotherapy (untreated) and patients with lymph node positive (LNP) colon cancer who received adjuvant chemotherapy were analysed separately to distinguish between the natural course of the disease (pure prognosis) and prognosis during adjuvant chemotherapy.

## Results

### Association of miRNA expression levels with clinical and histopathological characteristics

The total cohort comprised 232 patients: 155 patients with LNN primary colon cancer and 77 patients with LNP primary colon cancer. First, we assessed the distribution of mRNA expression for each miRNA and correlations between the expression levels of the miRNAs (Supplementary Fig. S1). The only miRNA for which expression did not follow a normal distribution was *miR-885-5p* (P < 0.001). All miRNAs significantly positively correlated with three or more of the six assessed miRNAs. However, only poor to moderate associations were observed (Spearman’s rho 0.13–0.33, P < 0.001–P = 0.044; Supplementary Fig. S1). Next, possible associations between miRNA expression and clinical and histopathological characteristics were assessed. Importantly, most of the significant differences in miRNA expression in the total group were derived from differences in the LNN group and not the smaller LNP group. As we were primarily interested in the association with pure disease prognosis, the LNN subgroup was the main focus of further analyses. The associations between clinical and histopathological features of the LNN group are shown in Table 1, the total group in Supplementary Table S1a, and the LNP group in Supplementary Table S1b.

**Table 1:** Clinical and histopathological characteristics of the LNN patients.

		N (%)	*miR-320*	P value	*miR-221*	P value	*miR-30b*	P value		P value	*miR-885–* *5p*	P value	*let-7i*	P value
*Gender*	Female	73 (47.1%)	−4.50(−5.01–4.10)	0.94	−1.78(−2.56–1.18)	0.42	−1.79(−2.37–1.27)	0.025	−2.55(−3.12–1.80)	0.09	−11.20(−13.00–10.32)	0.96	−2.97(−3.45–2.28)	0.14
	Male	82 (52.9%)	−4.55(−4.97–3.99)		−1.77(−2.39–0.97)		−1.59(−1.99–1.14)		−2.68(−3.18–2.15)		−11.39(−12.74–10.48)		−3.16(−3.87–2.48)	
*Age*		155 (100%)	−0.05	0.57	−0.13	0.10	0.01	0.87	0.09	0.25	0.02	0.78	0.07	0.39
*Tumour stage*	Stage I	57 (36.8%)	−4.61(−5.01–4.03)	1.00	−1.78(−2.45–0.88)	0.65	−1.55(−1.97–1.07)	0.13	−2.62(−3.29–1.90)	0.96	−11.21(−12.88–10.09)	0.16	−2.59(−3.55–2.09)	0.037
	Stage II	98 (63.2%)	−4.49(−4.99–4.08)		−1.77(−2.51–1.16)		−1.75(−2.17–1.35)		−2.66(−3.13–2.02)		−11.39(−13.00–10.52)		−3.15(−3.61–2.66)	
*T status*	T2	57 (36.8%)	−4.61(−5.01–4.03)	1.00	−1.78(−2.45–0.88)	0.65	−1.55(−1.97–1.07)	0.13	−2.62(−3.29–1.90)	0.96	−11.21(−12.88–10.09)	0.16	−2.59(−3.55–2.09)	0.037
	T3	98 (63.2%)	−4.49(−4.99–4.08)		−1.77(−2.51–1.16)		−1.75(−2.17–1.35)		−2.66(−3.13–2.02)		−11.39(−13.00–10.52)		−3.15(−3.61–2.66)	
*Nodal status*	N0	127 (81.9%)	−4.47(−4.95–4.01)	0.23	−1.77(−2.51–1.13)	0.72	−1.75(−2.13–1.32)	0.053	−2.61(−3.11–1.91)	0.15	−11.34(−13.00–10.48)	0.56	−3.05(−3.54–2.38)	0.48
	N0 < 10 nodes	28 (18.1%)	−4.72(−5.14–4.13)		−1.71(−2.46–0.67)		−1.40(−1.99–0.91)		−2.73(−3.37–2.06)		−11.28(−12.94–9.50)		−3.12(−3.96–2.36)	
*Tumour grade*	Good	13 (8.4%)	−4.38(−4.91–3.99)	0.77	−1.59(−1.97–1.06)	0.21	−1.54(−1.95–1.26)	0.95	−2.74(−3.29–1.89)	0.98	−11.61(−13.00–10.50)	0.28	−3.01(−3.86–2.39)	0.51
	Moderate	130 (83.9%)	−4.52(−5.02–4.03)		−1.82(−2.50–1.14)		−1.74(−2.13–1.26)		−2.65(−3.13–1.95)		−11.32(−13.00–10.43)		−3.12(−3.56–2.36)	
	Poor	9 (5.8%)	−4.58(−5.41–4.22)		−2.35(−3.58–1.21)		−1.73(−2.58–1.01)		−3.00(−3.27–2.31)		−11.64(−12.91–10.60)		−2.87(−3.66–2.44)	
	Other	3 (1.9%)	−4.27(−4.71–)		−1.77(−2.16–)		−1.08(−1.52–)		−1.59(−2.26–)		−9.15(−10.49–)		−2.55(−3.00–)	
*Location*	Right	79 (51.0%)	−4.49(−4.88–4.09)	0.76	−2.16(−2.69–1.47)	0.001	−1.82(−2.16–1.46)	0.029	−2.49(−2.77–1.78)	<0.001	−11.22(−13.00–10.41)	0.70	−3.16(−3.59–2.37)	0.55
	Left	76 (49.0%	−4.58(−5.11–4.02)		−1.48(−2.19–1.09)		−1.56(−1.97–1.09)		−2.94(−3.41–2.29)		−11.35(−12.79–10.45)		−3.03(−3.54–2.44)	
*MSI-status* ^*a*^	MSI	34 (22.1%)	−4.50(−4.80–4.00)	0.44	−2.53(−3.06–2.10)	<0.001	−2.00(−2.20–1.60)	0.007	−2.12(−2.63–1.45)	<0.001	−12.33(−13.00–10.58)	0.011	−3.01(−3.34–2.46)	0.50
	MSS	120 (77.9%)	−4.51(−5.08–4.05)		−1.61(−2.30–0.94)		−1.61(−1.99–1.15)		−2.73(−3.24–2.12)		−11.21(−12.65–10.26)		−3.08(−3.68–2.35)	

Data are given as median (interquartile range). ^a^One missing value.

Expression of *miR-221*, *miR-30b*, and *miR-885–hyp5p* was significantly lower, whereas expression of *miR-10b* was significantly higher in MSI tumours than MSS tumours (Table 1). Expression of *miR-221* and *miR-30b* was significantly lower and *miR-10b* significantly higher in left-sided tumours than right-sided tumours. In addition to the associations of miRNAs with these two clinically important characteristics, expression of *let-7i* was significantly lower in stage II tumours than stage I tumours.

### *let-7i*, *MiR-30b*, and miRNA signature as prognostic markers of clinical outcome

The prognostic value of the six miRNAs was assessed using expression as a continuous variable in a univariate Cox regression model (Table 2). *Let-7i* expression was significantly associated with hepatic metastasis free survival (HFS) (hazard ratio [HR] = 0.32, 95% confidence interval [CI] = 0.17–0.60, P < 0.001). In contrast to the findings reported by Hur *et al*., *miR-10b* was not significantly associated with any of the clinical endpoints. However, the expression of *miR-30b* was significantly associated with metastasis free survival (MFS) and HFS (HR = 2.13, 95% CI = 1.22–3.72, P = 0.008 and HR = 2.77, 95% CI = 1.24–6.18, P = 0.013, respectively). None of the other miRNAs were associated with MFS or HFS, and none of the miRNAs were significantly associated with overall survival (OS).

**Table 2:** Univariate and multivariate Cox regression analysis for the LNN group.

		N (%)	Univariate						Multivariate					
			MFS (25 events)		HFS (12 events)		OS (23 events)		MFS (25 events)		HFS (12 events)		OS (23 events)	
			HR (95% CI)	P value	HR (95% CI)	P value	HR (95% CI)	P value	HR (95% CI)	P value	HR (95% CI)	P value	HR (95% CI)	P value
*mRNA expression*	*miR-320*	155 (100%)	0.80(0.49–1.31)	0.38	0.64(0.32–1.26)	0.20	0.86(0.51–1.47)	0.59						
	*miR-221*	155 (100%)	1.37(0.94–1.98)	0.098	0.91(0.53–1.57)	0.75	0.83(0.55–1.26)	0.38						
	*miR-30b*	155 (100%)	2.13(1.22–3.72)	0.01	2.77(1.24–6.18)	0.013	1.39(0.74–2.59)	0.31						
	*miR-10b*	155 (100%)	0.99(0.61–1.61)	0.97	0.70(0.35–1.38)	0.30	0.99(0.59–1.65)	0.97						
	*miR-885–5p*	155 (100%)	0.90(0.68–1.18)	0.44	0.84(0.55–1.27)	0.40	0.80(0.59–1.09)	0.15						
	*let-7i*	155 (100%)	0.69(0.45–1.07)	0.10	0.32(0.17–0.60)	<0.001	0.94(0.54–1.30)	0.43						
*Gender*	Female	73 (47.1%)	1		1		1							
	Male	82 (52.9%)	1.41(0.63–3.14)	0.40	1.84(0.55–6.11)	0.32	2.23(0.92–5.42)	0.08						
*Age*		155 (100%)	1.004(0.96–1.05)	0.88	1.01(0.94–1.09)	0.75	1.08(1.02–1.14)	0.01						
*Tumour stage*	Stage I	57 (36.8%)	1		1		1							
	Stage II	98 (63.2%)	1.51(0.63–3.61)	0.36	1.17(0.35–3.90)	0.79	1.07(0.45–2.53)	0.88						
*T status*	T2	57 (36.8%)	1		1		1							
	T3	98 (63.2%)	1.51(0.63–3.61)	0.36	1.17(0.35–3.90)	0.79	1.07(0.45–2.53)	0.88						
*Nodal status*	N0	127 (81.9%)	1		1		1		1		1		1	
	N0 < 10 nodes	28 (18.1%)	1.20(0.45–3.20)	0.72	3.42(1.08–10.76)	0.04	1.51(0.59–3.86)	0.39	1.08(0.40–2.88)	0.88	2.91(0.92–9.19)	0.07	1.44(0.55–3.75)	0.45
*Tumour grade*	Good	13 (8.4%)	1		1		1							
	Moderate	130 (83.9%)	1.01(0.24–4.31)	0.99	0.91(0.12–7.19)	0.93	1.98(0.27–14.80)	0.51						
	Poor	9 (5.8%)	2.12(0.35–12.69)	0.41	2.85(0.26–31.39)	0.39	4.47(0.46–43.17)	0.20						
	Other^b^	3 (1.9%)	—		—		—							
*Location*	Right	79 (51.0%)	1		1		1							
	Left	76 (49.0%)	1.91(0.84–4.32)	0.12	1.08(0.35–3.33)	0.90	0.60(0.25–1.40)	0.24						
*MSI-status* ^***a***^	MSI	34 (22.1%)	1		1		1							
	MSS	120 (77.9%)	3.46(0.82–14.70)	0.09	30.43(0.1–9228.65)	0.24	0.48(0.21–1.11)	0.09						
*Modified signature*	low risk	79 (51.0%)	1		1		1		1		1		1	
	high risk	76 (49.0%)	2.73(1.14–6.55)	0.02	11.19(1.45–86.69)	0.02	1.31(0.57–2.99)	0.52	2.72(1.13–6.53)	0.03	10.30(1.33–79.992)	0.03	1.24(0.53–2.87)	0.62

^a^One missing value; ^b^there were no events in this subgroup.

**Figure 1:**
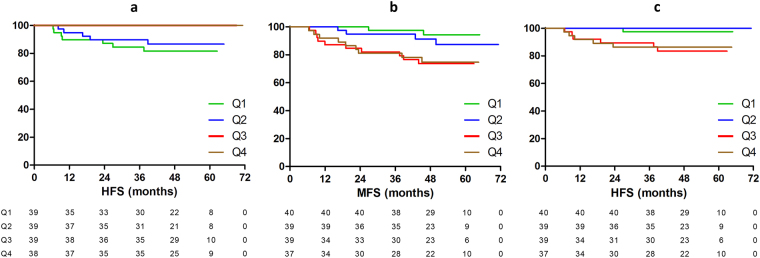
Kaplan Meier estimates for the correlation between *let-7i* with HFS and *miR-30b* with MFS and HFS in the LNN group. Q1 = lowest quartile, Q4 = highest quartile.

**Figure 2:**
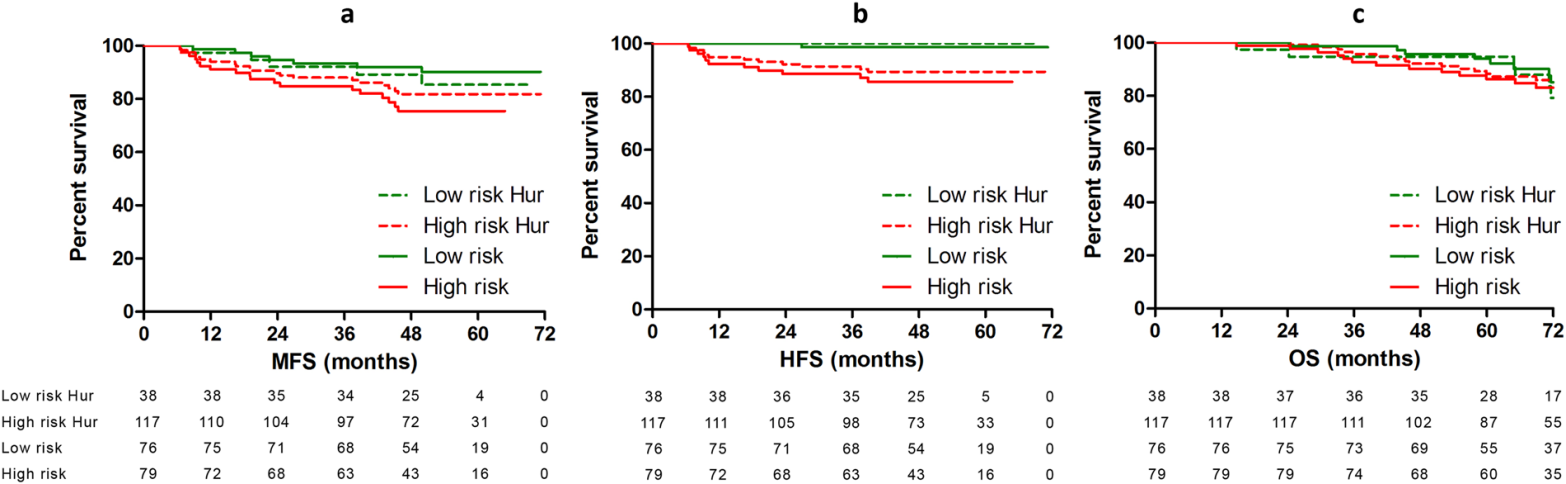
Kaplan Meier estimates for the correlation between the original metastasis-specific signature (dashed line) and modified metastasis-specific signature (continuous line) for MFS, HFS, and OS.

The significant associations were visualized by Kaplan Meier analysis dividing the expression levels of *let-7i* and *miR-30b* into quartiles (Fig. 1), showing a split course for Q1–2 (below median) and Q3–4 (above median). The median expression levels were used to assess the prognostic value of the original 2-miRNA signature in our cohort (*let-7i* high and *miR-10b* low vs. *let-7i* low and/or *miR-10b* high). The 2-miRNA signature was significantly associated with HFS (5-year survival 100% vs. 89.3%, P = 0.04; Fig. 2a) but did not demonstrate a significant difference for MFS (Fig. 2b) or OS (Fig. 2c).

As *miR-30b* was significantly associated with MFS and HFS (Fig. 1b and 1c, respectively) in our cohort, we explored whether *miR-30b* could contribute to the discriminating value of the signature. In a multivariate Cox regression model including the 2-miRNA signature and *miR-30b* expression split at the median level, *miR-30b* was significantly associated with MFS (HR = 3.65, 95% CI = 1.44–9.27, P = 0.007) and HFS (HR = 10.04, 95% CI = 1.30–77.78, P = 0.027) independent of the original signature. For both models, the log likelihood significantly increased when adding *miR-30b* expression split at the median level (Δ log likelihood = 4.36, P = 0.003 and Δ log likelihood = 4.43, P = 0.003). Therefore, we added the *miR-30b* split at the median expression level to the original metastasis-specific miRNA signature (modified 3-miRNA signature), in which patients were categorized as low risk when having a tumour with at least two of the following: *let-7i* high, *miR-10b* low, and *miR-30b* low. All other patients were categorized as high risk. The modified 3-miRNA signature was significantly different in regards to MFS (5-year survival 90.2% vs. 75.5%, P = 0.019) and HFS (5-year survival 98.6% vs. 85.6%, P = 0.004; Figs 2a and b, respectively). In the total group, this modified signature also demonstrated a significant difference between patients in the ‘low risk’ and ‘high risk’ groups for both MFS (5-year survival 86.8% vs. 73.8%, P = 0.018) and HFS (5-year survival 97.0% vs. 86.2%, P = 0.004). However, the modified 3-miRNA signature did not correlate with OS in these groups or with any of the clinical endpoints in the LNP group.

Nodal status was the only traditional clinical factor significantly associated with disease outcome in the univariate Cox regression analysis in both the LNN and total groups (Table 2 and Supplementary Table S2, respectively). When adjusting for lymph node status in a multivariate Cox regression model, which was stratified in the LNN group into two groups based on the total number of assessed lymph nodes (cut-off = 10), the modified metastasis-specific 3-miRNA signature was still significantly associated with MFS (HR = 2.72, 95% CI = 1.13–6.53, P = 0.025) and HFS (HR = 10.30, 95% CI = 1.33–79.99, P = 0.026) in the LNN group and HFS in the total group (HR = 5.07, 95% CI = 1.46–17.59, P = 0.011; Table 2 and Supplementary Table S2, respectively).

### Pathway analysis

Pathway analysis showed that *let-7i* expression was associated with axon guidance, glycosphingolipid and glycosaminoglycan biosynthesis, focal adhesion, extracellular matrix (ECM) receptor interaction, and regulation of the actin cytoskeleton, which are all related to cell adhesion, migration, and motility. Furthermore, we observed an association with the hedgehog, WNT, and transforming growth factor (TGF)-β signalling pathways (Supplementary Tables S3 and S4). Combined use of three independent target prediction algorithms did not reveal any overlapping *let-7i* target genes involved in glycosphingolipid biosynthesis, glycosaminoglycan biosynthesis, or the hedgehog signalling pathway. However, the axon guidance, focal adhesion, ECM receptor interaction, regulation of actin cytoskeleton, TGF-β signalling, and WNT signalling pathways do contain a number of genes predicted to be *let-7i* targets by all three algorithms (Supplementary Table S5). The expression of *COL4A6* and *FNDC3A*, which are involved in focal adhesion and ECM receptor interactions, significantly negatively correlated at the mRNA level to *let-7i* expression in the LNN and LNP samples. Expression of *ACVR1C*, part of the TGF-β signalling pathway, negatively correlated with *let-7i* only in LNP samples (P = 0.056).

Expression of *miR-30b* was associated with axon guidance and significantly negatively correlated with the expression of *PPP3R1, NFAT5*, and *SEMA6B* in this pathway (Supplementary Tables S3–5).

Notably, we observed more significant positive correlations between *let-7i* expression and its predicted targets in all significantly associated pathways, suggesting that the effect of *let-7i* on genes in these pathways are mostly indirect.

## Discussion

Our study confirmed the clinical significance of measuring *let-7i* and the miRNA-signature as suggested by Hur *et al*. and have extended these findings to a well-defined independent cohort of patients with colon cancer. We showed that the expression of most of these miRNAs is different for MSI and MSS tumours and for left- and right-sided tumours. Furthermore, let-7i was expressed at a lower level in stage II colon cancer compared to stage I colon cancer. We validated *let-7i* as a prognostic marker but could not confirm *miR-10b* as a prognostic factor. In contrast, *miR-30b* was prognostic with regard to MFS and HFS. The original metastasis-specific 2-miRNA signature was significantly associated with HFS but not MFS. Therefore, we propose a modified metastasis-specific 3-miRNA signature combining *miR-10b, miR-30b*, and *let-7i*, which identified groups with low and high risk in terms of MFS and HFS. Subsequent pathway analysis suggested an association between *let-7i* expression and cell adhesion, migration, and motility, and the hedgehog, WNT, and TGF-β signalling pathways. *miR-30b* expression was associated with axon guidance.

The findings with regard to differential expression of the miRNAs in MSI versus MSS and left-sided versus right-sided tumours suggest a different role of these miRNAs in hypermutated and non-hypermutated tumours, adding to our understanding of these biologically different entities. Although these characteristics were not included in the differential miRNA expression analysis by Hur *et al*., the findings are in line with accumulating evidence of the role of miRNAs in the pathogenesis of MSI tumours, such as the involvement of *miR-21* and *miR-155* in the regulation of mismatch repair gene and protein expression^10^. Similarly, left- and right-sided tumours are reported to express miRNAs at different levels^11,12^. Our findings add to the fast expanding knowledge on the different roles of miRNAs in these biologically different entities. Furthermore, we found that *let-7i* expression was lower in stage II tumours compared to stage I tumours, which is line with Hur *et al*.’s findings and the tumour suppressing role of the *let-7* family as described in the literature^13^.

Furthermore, low expression of *let-7i* was associated with poor HFS in our cohort of patients with LNN colon cancer who did not receive adjuvant chemotherapy, which confirmed the findings of Hur *et al*. We also found that high expression of *miR-30b* was associated with poor MFS and HFS, which was not observed by Hur *et al*., because *miR-30b* was not validated in the comparative analysis of primary CRC and matched metastatic liver tissues used for the identification of miRNAs that may hold prognostic potential in primary tumours. In our cohort, all patients had colon cancer, which may explain the different observations in our cohort. In contrast to Hur *et al*., we did not find a significant association or trend between the expression of *miR-10b* and any of the long-term clinical endpoints. Interestingly, Hur *et al*. showed that *miR-10b* is downregulated in metastatic liver tissue compared to primary CRC tissue, whereas it is upregulated in primary CRC versus normal tissue, and high expression of *miR-10b* is associated with poor MFS. This suggests that differential expression between primary and metastatic lesions does not always reflect the prognostic and/or predictive value in primary tumour lesions^14–19^. This may also explain the discrepancy in *miR-30b* between the absence of differential expression in their comparative analysis and the association with MFS/HFS in our cohort, although further studies are needed to confirm the latter.

Hur *et al*. also showed that their signature, consisting of *miR-10b* and *let-7i*, was associated with MFS in one cohort of primary colorectal cancers. Although this signature segregated our cohort of patients with LNN colon cancer into two groups with significantly different outcomes in terms of HFS, we did not find a significant difference in terms of MFS. Incorporation of *miR-30b* (modified signature) identified low and high risk groups that were clearly distinctive with regard to MFS and HFS. These results remained significant after correcting for nodal status. Next to nodal status, MSI was significantly associated with HFS in the LNN group. Furthermore, when correcting for MSI alone or together with nodal status in the LNN group, the modified metastasis-specific signature remained significantly associated with MFS and HFS (data not shown). However, due to the low number of events, these multivariable analyses should be interpreted with caution. Taken together, our results underline the prognostic value of *let-7i* and provide support for the prognostic value of the modified miRNA signature.

The different observations can be explained by differences in the patient cohorts used in the study by Hur *et al*. and the current study. The cohorts used by Hur *et al*. were heterogeneous with regard to tumour stage and tumour location (both colon and rectum were included), and no information was provided on pre-operative and postoperative local and systemic treatment^9^. This heterogeneity may explain, for example, the failure of validating miR-30b in the comparative analysis, as *miR-30b* expression varies between colon and rectal cancers^12^; a well-defined prospective cohort of patients with colon cancer was used in the current study. Another explanation for the observed differences may be the cut-off used to dichotomize expression levels. Hur *et al*. used Youden’s index (i.e., the most optimal combination of sensitivity and specificity) to determine the cut-off value for the maximum potential effectiveness of miRNAs based on a specific outcome parameter^20^. Inherent to the dependency of the cut-off to the outcome parameter, patients may change from low risk to high risk depending on the outcome of interest. In our cohort, the cut-off value based on Youden’s index was nearly identical to the median expression level, which is independent of the outcome parameter of interest and, per definition, gives two equally sized subgroups (data not shown). In their cohort, Youden’s index stratified the patients into two unequally sized groups (4.8% low *let-7i* expression and 21% low *miR-10b* expression); thus, the cut-off value based on Youden’s index was quite different from the median expression level. Therefore, future studies should explore the median expression level to assign patients to a low or high risk group. Lastly, differences in the material used (formalin-fixed paraffin embedded versus fresh frozen), RNA isolation, and/or assays used to measure miRNA expression may account for part of the observed differences.

The pathway analysis for *let-7i* and *miR-30b* revealed several interesting associations. *Let-7i* expression was associated with several pathways related to cell adhesion, migration, and motility. This is in line with the literature, which indicates that the expression of this miRNA is associated with the expression of genes in these pathways that are to known to be altered in carcinogenesis and involved in progression^21,22^. Activation of either LIN28A or LIN28B is thought to be responsible for global post-transcriptional downregulation of the *let-7* family in cancer^23^. Furthermore, *let-7i* expression was associated with the hedgehog, WNT, and TGF-β signalling pathways. Interestingly, the *let-7* family was shown to be involved in hedgehog-mediated drug resistance in lung cancer, which supports the observed association in our cohort^24^. Similarly, the *let-7* family was shown to be involved in Wnt signalling in breast cancer, which again involves LIN28^25^. Lastly, high expression of *let-7i* was associated with increased TGF-β signalling, which plays a major role in the tumourigenesis of at least half of all CRCs in which inactivating mutations abolish the tumour suppressing effect of the TGF-β signalling pathway. Furthermore, decreased SMAD4 expression, a downstream target of TGF-β, is associated with poor prognosis in colon cancer, providing indirect evidence that inactivation of TGF-β signalling leads to the invasive behaviour of colon cancer^26^. Interestingly, TGF-β appears to convert from a tumour suppressor to a tumour promotor in more advanced stages of cancer, which is known as the TGF-β paradox^27^. A few studies have specifically addressed the association between let-7 and TGF-β expression, showing an inverse correlation^28,29^. However, these studies were performed in cell lines and a melanoma xenograft model, which may preclude extrapolation of these results to CRC in a clinical setting. Using three prediction algorithms, 6% of the genes in the TGF-β signalling pathway were considered a potential target of *let-7i*. The positive correlation between tumour suppressor *let-7i* and genes involved in the TGF-β signalling pathway suggests that TGF-β has yet to go through this conversion, and both TGF-β and let-7i act as tumour suppressors. Interestingly, expression of *ACVR1C* was negatively associated with *let-7i* expression in only the LNP group, suggesting that these tumours may have gone through the conversion. The association with the above-mentioned pathways may also suggest that *let-7i* is linked to stromal content and plays an indirect role in TGF-β signalling in stromal cells, which is involved in in metastasis initiation^30^.

The miR-30 family has been studied extensively in the field of cancer research. *In vitro*, miR-30 family members have been associated with several aspects of tumourigenesis, including cell migration, cell growth, cell invasiveness, and apoptosis^31–39^. Interestingly, both negative and positive associations with tumourigenesis have been described for miR-30 family members, including miR-30b, which precludes definitive conclusions. Contrasting associations have also been reported between the miR-30 family and tumour characteristics, such as tumour stage and tumour grade^31,37,38,40–43^. In terms of clinical outcome, defined as MFS and/or OS, miR-30 family members have been described as markers of poor outcome in melanoma^40^, ovarian cancer^32^, prostate cancer^35^, and oesophageal cancer^44^, and as a tumour suppressive miRNA in breast cancer^42^, lung cancer^45^, CRC^33^, prostate cancer^31^, and ovarian cancer^46^. In our study, high *miR-30b* expression was associated with poor MFS and HFS. The pathway analysis mainly revealed a positive association between *miR-30b* expression and axon guidance. The genes traditionally described by their roles in axon guidance are important regulators of neuronal migration and positioning during embryonic development. However, they have been implicated in cancer cell survival, growth, invasion, and angiogenesis^47,48^. Very little is available in the current literature on the direct association between *miR-30b* and axon guidance. However, the target gene prediction we performed subsequent to the pathway analysis predicted that >11% of the genes listed in the ‘axon guidance’ pathway in KEGG were direct targets of *miR-30b*. In support of this direct interaction, significant negative correlations between the expression of *miR-30b* and axon-guidance genes *PPP3R1, NFAT5*, and *SEMA6B* were observed in our cohort. Further research may be directed to investigate the possible role of *miR-30b* in axon guidance. These insights, combined with the fact that miR-30b was upregulated in patients in our cohort with a poor prognosis, may provide a rationale for investigating the miRNA as a therapeutic target.

Although overlapping predicted targets were found for the majority of *let-7i-* and *miR-30b*-associated pathways in three prediction algorithms, we observed many significantly positive correlations between *let-7i* and its predicted targets in our cohort. Thus, the effects of *let-7i* on the associated pathways are (partially) indirect, and functional studies are needed to confirm this. Overall, the observed associations suggest that *let-7i* and *miR-30b* may be relevant factors for cancer cells in their ability to move, potentially also involving their stromal component, increasing their metastatic potential.

Our data suggest that *let-7i*, *miR-30b*, and a three-miRNA signature hold prognostic value in LNN colon cancers, although independent validation in a large cohort is needed. Ideally, further studies should include an analysis of the circulating serum levels of these miRNAs.

## Methods

All aspects of the guidelines for REporting recommendations for tumour MARKer (REMARK) prognostic studies were followed, and the paper was written accordingly^49^. The study was conducted according to the Declaration of Helsinki. All procedures involving human subjects were approved by the Erasmus MC University Medical Centre Institutional Review Board (MEC 2007-088).

### Patient selection

Patients were selected from the MATCH cohort, an observational prospective multicentre cohort study conducted from 2007 onwards that includes patients undergoing curative surgery for CRC in one of seven participating hospitals in the Rotterdam region of the Netherlands. Patients provided written informed consent for the storage and use of biobank samples for research purposes and the collection of clinical data (Institutional Review Board number MEC 2007-088).

**Figure 3:**
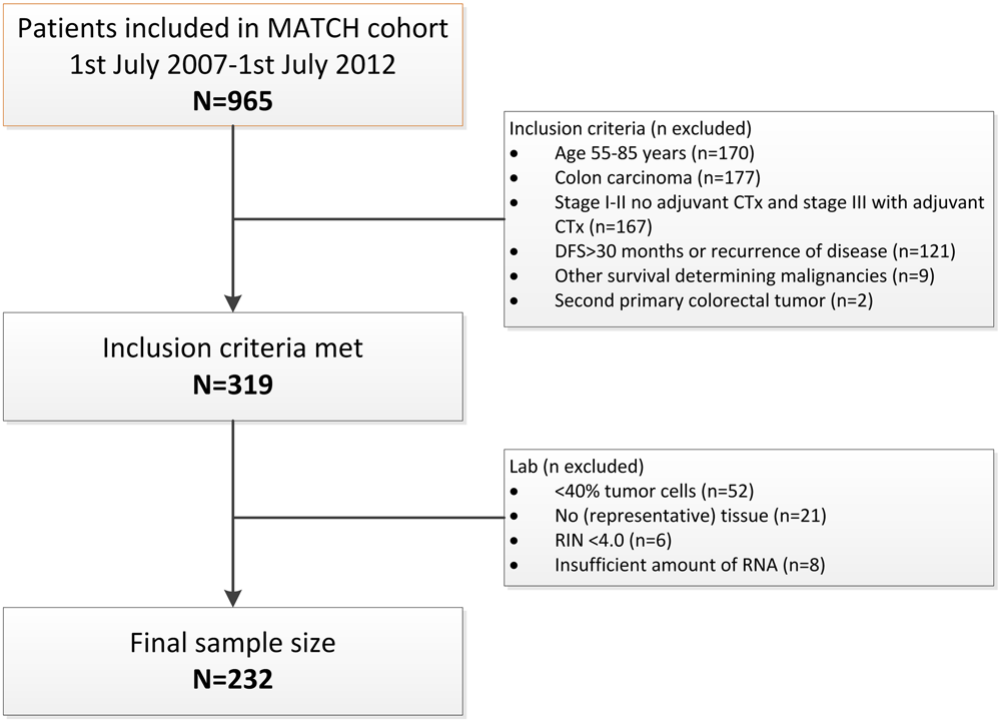
Diagram of patient selection.

Additional inclusion criteria for this study were: inclusion date between 1 July 2007 and 1 July 2012, age 55–85 years, stage I-II colon cancer without adjuvant chemotherapy or stage III colon cancer with adjuvant chemotherapy, fresh frozen tissue with at least 40% invasive tumour cells available, and either recurrence of disease or at least 30 months of disease-free follow-up (Fig. 3).

### Sample collection and processing

Resected specimens from the primary tumours were transported to the pathology laboratory immediately following removal during surgery. Two to four samples of both the central and peripheral regions of the tumour and one or two adjacent non-tumour colon tissue samples were taken. The samples were fresh frozen with a maximum cold ischemia time of 2 hours. All samples were stored in liquid nitrogen.

### RNA isolation, cDNA synthesis, and mRNA transcript quantification

A cryostat microtome set at −20 °C was used to cut the fresh frozen colon cancer and normal colon tissues (Thermo Scientific Microm HM 560, Thermo Fisher Scientific, Inc.). A 5-µm section was cut before, during, and after sectioning for RNA isolation. After haematoxylin-eosin (HE) staining, the sections were reviewed independently by two pathologists. The percentage of neoplastic cells, infiltrating immune cells, necrosis, and normal mucosa was scored in categories of 0–5%, 6–10%, 11–20%, 21–30%, 31–40%, 41–50%, 51–60%, 61–70%, 71–80%, 81–90%, and 91–100% relative to other cells. Tumour classification and grading was determined using the WHO 2010 classification for carcinoma of the colon and rectum (WHO Press, World Health Organization, 20 Avenue Appia, Geneva, Switzerland).

Total RNA was isolated from 30-µm sections using RNA-Bee® according to the manufacturer’s instructions (Tel-Test, Inc., USA). The quality and quantity of the RNA was assessed using the MultiNA Microchip Electrophoresis system (Shimadzu, Kyoto, Japan) and Nanodrop ND-1000 (Thermo Scientific, Wilmington, USA), respectively.

The expression of the six miRNAs was quantified in a 9-plex miRNA assay protocol utilizing commercially available and validated TaqMan miRNA assays (Applied Biosystems, Thermo Scientific, USA) relative to the average of reference genes *miRNA-16*, *RNU6B*, and *RNU44* in the same total RNA sample.

Briefly, 5 μL samples containing 20 ng/μL total RNA were reverse-transcribed for 30 min at 16 °C, 30 min at 42 °C, 5 min at 85 °C, and stopped at 4 °C in the presence of 18 nM of each of the nine TaqMan RT-primers in 1x RT buffer (Fermentas) supplemented with 0.65 mM of each dNTP (Fermentas), 0.25 U/μL RNAseout (Fermentas), 3.8 mM MgCl_2_ (Invitrogen) in the absence (negative control) or presence of 15 U/μL revertAid MLV H-minus RT (Fermentas). After the reverse transcription, the samples were diluted 20-fold in LoTE (3 mM Tris-HCl/0.2 mM EDTA, pH 8.0) prior to 40 cycles of individual qPCR reactions for each of the nine miRNA assays in the presence of TaqMan Master mix without UNG as advised by the manufacturer (Applied Biosystems, Thermo Scientific, USA). The specifics of the six target miRNA assays and three reference miRNA assays used to normalize the data are provided in Supplementary Table S6.

### Microsatellite instability

Genomic DNA was extracted from 2 to 5 × 30 µm sections cut from between the sections used for RNA isolation (NucleoSpin Tissue kit, Macherey-Nagel, BIOKE, Leiden, the Netherlands). MSI status was determined by a fluorescent PCR-based assay in five mononucleotide repeat markers (BAT-25, BAT-26, NR-21, NR-24, and MONO-27; Promega MSI Analysis System) using 2 ng of PicoGreen-measured DNA. Quality and quantity were assessed by agarose gel electrophoresis, Nanodrop, and the Quant-iT PicoGreen dsDNA kit (Life Technologies). Two pentanucleotide repeat markers (Penta C and Penta D) were also included to detect potential sample mix-ups and/or contamination using the manufacturer’s protocol.

### Pathway analysis

RNA sequencing data were available for 231 patients^50^. We used the STAR^51^ algorithm (version 2.4.2a) to align the RNA-seq data with the GRCh38 reference using the ‘–quantmode Genecounts’ option to obtain the raw read counts for each gene. Gene annotation was derived from gencode v23 (https://www.gencodegenes.org/). Next, the trimmed mean of M-values normalization^52^, as implemented in EdgeR^53^, was used to normalize the raw read count data. These data were used as the input in the pathway analysis.

For the miRNAs associated with long-term clinical outcomes in our cohort, the 50 tumours with the highest expression and 50 tumours with the lowest expression of each miRNA were grouped and used as input. Pathway analyses were performed using the R-package ‘global test’ with KEGG^54^. Importantly, only genes for which expression data were available were used as input. The Bonferroni-Holm method was used to correct all P values for multiple testing. Re-sampling (n = 1,000) was performed to determine the number of times a randomly selected group of genes of equal size was at least as significant as the true set of genes assigned to a pathway. The gene plots of pathways with a corrected P value < 0.05 and a re-sampling probability < 0.05 were reviewed. Three target gene prediction databases were used to identify genes within the selected pathways that were predicted to be potential targets of the respective miRNAs (Targetscan version 7.1, http://www.targetscan.org^55^; MicroRNA Target Prediction and Functional Study Database [miRDB], http://mirdb.org/miRDB^56^; RNA22 version 2.0, https://cm.jefferson.edu/rna22/^57^). We considered genes to be a potential target of an miRNA when predicted by all three databases and when the binding site was a conserved site within the 3′ UTR region.

### Survival data

MFS was defined as the time elapsed between the date of surgery and either the date of the appearance of distant metastasis or the date of the last follow-up visit at which a patient was considered to have no recurrence. HFS was defined as the time elapsed between the date of surgery and either the date of the appearance of liver metastasis or the date of the last follow-up visit at which a patient was considered to have no liver metastases. OS was defined as the time elapsed between the date of surgery and either the date of death or the date of the last check in the Municipal Personal Records Database.

### Statistical analysis

Statistical analyses were performed using SPSS statistical package version 21. Associations between the expression of the six miRNAs as continuous variables and clinical and histopathological characteristics were assessed using the Mann-Whitney U test, Spearman Rank correlation test, Kruskal-Wallis test, and Jonckheere-Terpstra test as appropriate. Cox regression analysis was used to assess the association between the expression of the six miRNAs as continuous variables and MFS, HFS, and OS. A one-way ANOVA was used to assess the difference between the two log likelihood estimates of the Cox regression models when adding a variable to the model. Kaplan Meier estimates were used to visualize the relevant associations between the miRNAs and long-term clinical outcome. Youden’s index was calculated as described previously^20^. All analyses were two-sided and P < 0.05 considered significant.

### Data availability

All data generated or analysed during this study are included in this published article and its Supplementary Information files. The RNA sequencing data were published previously and made available elsewhere^50^.

## Electronic supplementary material

Supplementary information file
