# Supplementary material for: Confirmation of a metastasis-specific microRNA signature in primary colon cancer
Source: Sci Rep. 2018 Mar 27;8:5242. doi: 10.1038/s41598-018-22532-1 (PMC5869672; doi:10.1038/s41598-018-22532-1)

Supplementary information to:

Confirmation of a metastasis-specific microRNA signature in primary colon cancer

Robert R.J. Coebergh van den Braak[a*], Anieta M. Sieuwerts[b,c], Zarina S. Lalmahomed[a], Marcel Smid[b], Saskia M. Wilting[b], Sandra I. Bril[a,b], Shanshan Xiang[a,b], Michelle Daane – van der Vlugt[b], Vanja de Weerd[b], Anne van Galen[b], Katharina Biermann[d], J Han JM van Krieken[e], Wigard P. Kloosterman[f], John A. Foekens[b], MATCH study group, John W.M. Martens[b,c], Jan N.M. IJzermans[a].

[a] Department of Surgery, Erasmus MC Medical Center, 's Gravendijkwal 230, 3015 CE Rotterdam, the Netherlands, [b] Department of Medical Oncology, Erasmus MC Cancer Institute, Erasmus University Medical Center, Rotterdam, the Netherlands, [c] Cancer Genomics Center Netherlands, Amsterdam, The Netherlands,[d] Department of Pathology, Erasmus MC Medical Center, Rotterdam, the Netherlands, [e] Department of Pathology, Radboud UMC, Geert Grooteplein Zuid 10, 6525 GA Nijmegen, the Netherlands, [f] Department of Genetics, Center for Molecular Medicine, University Medical Center Utrecht, Heidelberglaan 100, 3584 CX Utrecht, The Netherlands

Supplementary Table S1a. Clinical and histopathological characteristics of the total group

				miR-320		miR-221		miR-30b		miR-10b		miR-885-5p		let-7i	
		n	%	median (IQR)	P value	median (IQR)	P value	median (IQR)	P value	median (IQR)	P value	median (IQR)	P value	median (IQR)	P value
Gender	Female	104	44.8%	-4.52 (-5.07 • -4.10)	0.82	-1.84 (-2.49 • -1.05)	0.64	-1.67 (-2.12 • -1.19)	0.008	-2.49 (-3.07 • -1.90)	0.26	-11.10 (-13.00 • -10.16)	0.30	-2.88 (-3.44 • -2.37)	0.14
	Male	128	55.2%	-4.56 (-4.94 • -4.03)		-1.77 (-2.35 • -1.08)		-1.47 (-1.92 • -1.03)		-2.64 (-3.15 • -2.03)		-10.93 (-12.29 • -10.20)		-3.10 (-3.71 • -2.50)	
Age		232	100.0%	-0.04	0.52	-0.09	0.20	-0.07	0.26	.01	0.94	-0.03	0.70	0.03	0.61
Tumor stage	Stage I	57	24.6%	-4.61 (-5.01 • -4.03)	0.62	-1.78 (-2.45 • -0.88)	0.92	-1.55 (-1.97 • -1.07)	0.002	-2.62 (-3.29 • -1.90)	0.27	-11.21 (-12.88 • -10.09)	0.041	-2.59 (-3.55 • -2.09)	0.47
	Stage II	98	42.2%	-4.49 (-4.99 • -4.08)		-1.77 (-2.51 • -1.16)		-1.75 (-2.17 • -1.35)		-2.66 (-3.13 • -2.02)		-11.39 (-13.00 • -10.52)		-3.15 (-3.61 • -2.66)	
	Stage III	77	33.2%	-4.61 (-5.08 • -4.11)		-1.82 (-2.33 • -1.01)		-1.33 (-1.62 • -1.01)		-2.45 (-2.97 • -1.98)		-10.52 (-11.46 • -9.86)			
T status	T2	68	29.3%	-4.58 (-4.93 • -4.03)	0.61	-1.78 (-2.39 • -0.99)	0.52	-1.48 (-1.92 • -0.99)	0.33	-2.58 (-3.17 • -1.89)	0.93	-10.79 (-12.58 • -9.99)	0.20	-2.65 (-3.54 • -2.12)	0.054
	T3	164	70.7%	-4.51 (-5.05 • -4.10)		-1.80 (-2.37 • -1.13)		-1.59 (-1.97 • -1.14)		-2.59 (-3.08 • -2.01)		-11.11 (-13.00 • -10.26)		-3.09 (-3.56 • -2.62)	
Nodal status	N0	127	54.7%	-4.47 (-4.95 • -4.01)	0.38	-1.77 (-2.51 • -1.13)	0.48	-1.75 (-2.13 • -1.32)	<0.001	-2.61 (-3.11 • -1.91)	0.54	-11.34 (-13.00 • -10.48)	0.002	-3.05 (-3.54 • -2.38)	0.87
	N0 <10 nodes	28	12.1%	-4.72 (-5.14 • -4.13)		-1.71 (-2.46 • -0.67)		-1.40 (-1.99 • -0.91)		-2.73 (-3.37 • -2.06)		-11.28 (-12.94 • -9.50)		-3.12 (-3.96 • -2.36)	
	N1	52	22.4%	-4.69 (-5.06 • -4.16)		-1.89 (-2.32 • -1.15)		-1.32 (-1.64 • -0.92)		-2.46 (-3.13 • -2.03)		-10.62 (-11.49 • -9.89)		-2.86 (-3.39 • -2.46)	
	N2	25	10.8%	-4.41 (-5.19 • -3.83)		-1.42 (-2.35 • -0.52)		-1.38 (1.53 • -1.01)		-2.36 (-2.84 • -1.95)		-10.41 (-12.12 • -9.66)		-3.08 (-3.73 • -2.65)	
Tumor grade	Good	20	8.6%	-4.61 (-5.12 • -4.11)	0.41	-1.61 (-2.24 • -1.12)	0.72	-1.58 (-1.98 • -1.22)	0.056	-2.73 (-3.13 • -2.31)	0.56	-11.36 (-11.85 • -10.26)	0.14	-3.08 (-3.72 • -2.68)	0.34
	Moderate	184	79.3%	-4.55 (-5.05 • -4.04)		-1.81 (-2.40 • -1.06)		-1.58 (-1.99 • -1.16)		-2.58 (-3.11 • -1.95)		-11.11 (-13.00 • -10.24)		-3.06 (-3.56 • -2.44)	
	Poor	20	8.6%	-4.38 (-4.84 • -4.14)		-1.58 (-2.50 • -0.83)		-1.24 (-1.71 • -0.92)		-2.84 (-3.28 • -2.28)		-10.51 (-12.52 • -9.94)		-2.95 (-3.72 • -2.44)	
	Other	8	3.4%	-4.36 (-5.14 • -3.68)		-2.21 (-2.35 • -1.85)		-1.49 (-1.61 • -0.80)		-1.79 (-2.55 • -1.17)		-9.98 (-10.94 • -9.19)		-2.86 (-3.04 • -2.24)	
Location	Right	115	49.6%	-4.41 (-4.84 • -4.03)	0.036	-2.11 (-2.56 • -1.17)	0.006	-1.62 (-2.01 • -1.23)	0.016	-2.25 (-2.72 • -1.71)	<0.001	-11.10 (-13.00 • -10.10)	0.98	-2.99 (-3.55 • -2.38)	0.48
	Left	117	50.4%	-4.67 (-5.15 • -4.11)		-1.60 (-2.23 • -0.97)		-1.41 (-1.91 • -1.04)		-2.88 (-3.39 • -2.34)		-11.01 (-12.62 • -10.21)		-3.09 (-3.59 • -2.50)	
MSI-status [a]	MSI	46	19.8%	-4.38 (-4.86 • -3.90)	0.18	-2.45 (-3.05 • -2.10)	<0.001	-1.88 (-2.14 • -1.52)	<0.001	-2.12 (-2.59 • -1.64)	<0.001	-11.65 (-13.00 • -10.53)	0.001	-2.79 (-3.26 • -2.38)	0.09
	MSS	185	79.7%	-4.55 (-5.08 • -4.09)		-1.65 (-2.24 • -0.96)		-1.48 (-1.91 • -1.06)		-2.65 (-3.19 • -2.13)		-10.92 (-12.15 • -10.10)		-3.08 (-3.65 • -2.46)	

[a] n=1 missing

Supplementary Table S1b. Clinical and histopathological characteristics of the lymph node positive group

				miR-320		miR-221		miR-30b		miR-10b		miR-885-5p		let-7i	
		n	%	median (IQR)	P value	median (IQR)	P value	median (IQR)	P value	median (IQR)	P value	median (IQR)	P value	median (IQR)	P value
Gender	Female	31	40.3%	-4.73 (-5.18 • -4.11)	0.61	-2.10 (-2.37 • -0.79)	0.84	-1.47 (-1.77 • -1.06)	0.22	-2.46 (-2.97 • -2.13)	0.57	-11.03 (-13.00 • -10.06)	0.09	-2.80 (-3.39 • -2.56)	0.57
	Male	46	59.7%	-4.59 (-4.93 • -4.11)		-1.81 (-2.25 • -1.19)		-1.32 (-1.57 • -0.94)		-2.39 (-2.98 • -1.92)		-10.47 (-11.13 • -9.62)		-3.06 (-3.57 • -2.51)	
Age		77	100%	-0.06	0.60	0.01	0.91	-0.09	0.43	-0.15	0.19	-0.02	0.87	-0.05	0.70
Tumor stage	Stage I	0	0.0%	-	-	-		-		-		-		-	
	Stage II	0	0.0%	-	-	-		-		-		-		-	
	Stage III	77	100%	-4.61 (-5.08 • -4.11)		-1.82 (-2.33 • -1.01)		-1.33 (-1.62 • -1.01)		-2.45 (-2.97 • -1.98)		-10.52 (-11.46 • -9.86)		-2.99 (-3.52 • -2.52)	
T status	T2	11	14.3%	-4.52 (-4.83 • -3.90)	0.38	-1.71 (-2.20 • -1.02)	0.44	-1.20 (-1.37 • -0.74)	0.128	-2.41 (-2.97 • -1.66)	0.71	-10.23 (-10.52 • -9.47)	0.10	-3.04 (-3.20 • -2.45)	0.81
	T3	66	85.7%	-4.65 (-5.09 • -4.13)		-1.84 (-2.36 • -0.99)		-1.38 (-1.66 • -1.02)		-2.46 (-2.98 • -1.98)		-10.65 (-11.64 • -9.90)		-2.97 (-3.53 • -2.53)	
Nodal status	N0	0	0.0%	-	-	-		-		-		-		-	
	N0 <10 nodes	0	0.0%	-	-	-		-		-		-		-	
	N1	52	67.5%	-4.69 (-5.06 • -4.16)	0.38	-1.89 (-2.32 • -1.15)	0.44	-1.32 (-1.64 • -0.92)	0.13	-2.46 (-3.13 • -2.03)	0.71	-10.62 (-11.49 • -9.89)	0.10	-2.86 (-3.39 • -2.46)	0.81
	N2	25	32.5%	-4.41 (-5.19 • -3.83)		-1.42 (-2.35 • -0.52)		-1.38 (1.53 • -1.01)		-2.36 (-2.84 • -1.95)		-10.41 (-12.12 • -9.66)		-3.08 (-3.73 • -2.65)	
Tumor grade	Good	7	9.1%	-4.88 (-5.44 • -4.25)	0.11	-2.20 (-2.34 • -1.36)	0.57	-1.79 (-2.09 • -1.17)	0.11	-2.72 (-2.96 • -2.47)	0.52	-10.26 (-11.52 • -9.82)	0.61	-3.08 (-3.72 • -2.80)	0.50
	Moderate	54	70.1%	-4.69 (-5.07 • -4.09)		-1.80 (-2.36 • -0.99)		-1.32 (-1.60 • -0.99)		-2.35 (-2.94 • -1.89)		-10.74 (-11.79 • -10.01)		-2.92 (-3.53 • -2.49)	
	Poor	11	14.3%	-4.28 (-4.61 • -4.11)		-1.11 (-1.82 • -0.78)		-1.09 (-1.38 • -0.78)		-2.78 (-3.29 • -2.17)		-10.30 (-10.52 • -9.73)		-3.11 (-3.80 • -2.38)	
	Other	5	6.5%	-4.39 (-5.33 • -3.59)		-2.32 (-2.57 • -2.18)		-1.60 (-1.68 • -1.05)		-1.99 (-2.81 • -1.06)		-10.63 (-12.02 • -9.39)		-2.93 (-3.22 • -2.47)	
Location	Right	36	46.8%	-4.31 (-4.80 • -3.80)	0.00	-1.94 (-2.36 • -0.90)	0.96	-1.47 (-1.65 • -1.03)	0.36	-2.06 (-2.53 • -1.57)	<0.001	-10.54 (-11.39 • -9.62)	0.36	-2.78 (-3.11 • -2.39)	0.028
	Left	41	53.2%	-4.82 (-5.24 • -4.45)		-1.80 (-2.27 • -1.27)		-1.27 (-1.61 • -0.91)		-2.74 (-3.31 • -2.34)		-10.52 (-11.67 • -10.11)		-3.12 (-3.68 • -2.68)	
MSI-status	MSI	12	15.6%	-4.31 (-5.23 • -3.55)	0.33	-2.37 (-2.86 • -1.68)	0.015	-1.58 (-1.89 • -1.47)	0.009	-2.06 (-2.52 • -1.82)	0.049	-11.45 (-13.00 • -9.79)	0.11	-2.71 (-2.87 • -2.20)	0.018
	MSS	65	84.4%	-4.65 (-5.07 • -4.12)		-1.77 (-2.21 • -0.98)		-1.20 (-1.60 • -0.94)		-2.47 (-3.06 • -2.10)		-10.45 (-11.21 • -9.86)		-3.08 (-3.62 • -2.55)	

Supplementary Table S2. Univariate and multivariate Cox regression analysis for the total group

				Univariate						Multivariate					
				MFS (events=44)		HFS (events=19)		OS (events=41)		MFS (events=44)		HFS (events=19)		OS (events=41)	
		n	%	HR (95%CI)	P value	HR (95%CI)	P value	HR (95%CI)	P value	HR (95%CI)	P value	HR (95%CI)	P value	HR (95%CI)	P value
mRNA expression	*MiR-320*	232	100%	0.88 (0.60 • 1.30)	0.53	0.95 (0.53 • 1.71)	0.87	0.96 (0.64 • 1.45)	0.86						
	MiR-221	232	100%	1.36 (1.01 • 1.82)	0.041	1.24 (0.79 • 1.94)	0.35	1.02 (0.74 • 1.41)	0.89						
	MiR-30b	232	100%	1.93 (1.24 • 3.01)	0.004	2.37 (1.20 • 4.66)	0.013	1.35 (0.84 • 2.17)	0.22						
	MiR-10b	232	100%	1.03 (0.71 • 1.49)	0.87	0.65 (0.37 • 1.13)	0.13	1.02 (0.70 • 1.50)	0.92						
	MiR-885-5p	232	100%	0.98 (0.80 • 1.20)	0.85	0.89 (0.64 • 1.22)	0.45	0.94 (0.76 • 1.16)	0.57						
	Let-7i	232	100%	0.86 (0.62 • 1.19)	0.36	0.42 (0.27 • 0.64)	<0.001	0.92 (0.66 • 1.27)	0.60						
Gender	Female	104	44.8%	1		1		1							
	Male	128	55.2%	1.19 (0.65 • 2.18)	0.57	1.14 (0.46 • 2.82)	0.78	2.23 (1.15 • 4.59)	0.018						
Age		232	100%	1.0002 (0.96 • 1.04)	0.99	1.003 (0.95 • 1.06)	0.92	1.06 (1.02 • 1.11)	0.003						
Tumor stage	Stage I	57	24.6%	1		1		1							
	Stage II	98	42.2%	1.51 (.63 • 3.61)	0.36	1.17 (0.35 • 3.90)	0.79	1.08 (0.46 • 2.54)	0.87						
	Stage III			2.18 (0.92 • 5.18)	0.08	1.40 (0.41 • 4.79)	0.59	1.80 (0.78 • 4.15)	0.17						
T status	T2	68	29.3%	1		1		1							
	T3	164	70.7%	1.97 (0.92 • 4.24)	0.08	1.22 (0.44 • 3.38)	0.71	1.87 (0.86 • 4.07)	0.11						
Nodal status	N0	127	54.7%	1		1		1		1		1		1	
	N0 <10 nodes	28	12.1%	1.20 (0.45 • 3.20)	0.72	3.41 (1.08 • 10.75)	0.036	1.53 (0.6 • 3.88)	0.38	1.16 (0.43 • 3.08)	0.77	3.18 (1.01 • 10.02)	0.049	1.44 (0.56 • 3.68)	0.45
	N1	52	22.4%	0.99 (0.44 • 2.26)	0.99	1.79 (0.57 • 5.65)	0.32	1.01 (0.42 • 2.45)	0.98	0.90 (0.39 • 2.05)	0.80	1.43 (0.45 • 4.54)	0.54	0.94 (0.39 • 2.29)	0.89
	N2	25	10.8%	3.57 (1.71 • 7.47)	0.001	1.78 (0.37 • 8.59)	0.47	4.20 (1.96 • 9.00)	<0.001	2.93 (1.37 • 6.28)	0.006	1.19 (0.24 • 5.78)	0.83	3.60 (1.62 • 8.00)	0.002
Tumor grade	Good	20	8.6%	1		1		1							
	Moderate	184	79.3%	0.85 (0.30 • 2.39)	0.85	0.74 (0.17 • 3.26)	0.69	1.68 (0.40 • 7.04)	0.48						
	Poor	20	8.6%	1.70 (0.50 • 5.81)	0.40	1.50 (0.25 • 8.97)	0.66	4.17 (0.89 • 19.66)	0.07						
	Other^	8	3.4%	-		-		-							
						-		-							
Location	Right	115	49.6%	1		1		1							
	Left	117	50.4%	1.17 (0.65 • 2.12)	0.60	0.88 (0.36 • 2.17)	0.78	0.58 (0.31 • 1.09)	0.09						
MSI-status [a]	MSI	46	19.8%	1		1		1							
	MSS	185	79.7%	2.00 (0.79 • 5.08)	0.14	28.64 (0.24 • 3466.30)	0.17	0.64 (0.32 • 1.25)	0.19						
Modified signature	low risk	111	47.8%	1		1		1		1		1		1	
	high risk	121	52.2%	2.12 (1.12 • 4.00)	0.02	5.17 (1.51 • 17.76)	0.009	2.12 (1.12 • 4.00)	0.02	1.81 (0.94 • 3.50)	0.08	5.07 (1.46 • 17.59)	0.011	1.50 (0.76 • 2.96)	0.24

[a] n=1 missing

^ therer were no events in this subgroup

Supplementary Table S3. Pathway analysis results

	Let-7i		*MiR-30b*	
	P value	% of predicted genes	P value	% of predicted genes
GLYCOSPHINGOLIPID_BIOSYNTHESIS_GANGLIO_SERIES	1.35E-11	-		
GLYCOSAMINOGLYCAN_BIOSYNTHESIS_CHONDROITIN_SULFATE	1.95E-10	-		
AXON_GUIDANCE	3.41E-10	3.9%	3.59E-04	11.6%
FOCAL_ADHESION	7.61E-10	3.6%		
ECM_RECEPTOR_INTERACTION	1.33E-09	7.0%		
REGULATION_OF_ACTIN_CYTOSKELETON	1.80E-09	2.0%		
PATHWAYS_IN_CANCER	2.22E-09	-		
HEDGEHOG_SIGNALING_PATHWAY	2.44E-09	0.0%		
RENAL_CELL_CARCINOMA	2.72E-09	-		
DILATED_CARDIOMYOPATHY	4.69E-09	-		
HYPERTROPHIC_CARDIOMYOPATHY_HCM	4.83E-09	-		
WNT_SIGNALING_PATHWAY	9.16E-09	2.7%		
ARRHYTHMOGENIC_RIGHT_VENTRICULAR_CARDIOMYOPATHY_ARVC	1.05E-07	-		
TGF_BETA_SIGNALING_PATHWAY	2.73E-07	6.0%		
MELANOMA	4.10E-07	-		
GLYCOSPHINGOLIPID_BIOSYNTHESIS_GLOBO_SERIES	3.05E-06	-		
UBIQUITIN_MEDIATED_PROTEOLYSIS			3.90E-04	-
MELANOGENESIS			1.35E-03	-
RNA_POLYMERASE			2.58E-03	-
HOMOLOGOUS_RECOMBINATION			2.69E-03	-
SNARE_INTERACTIONS_IN_VESICULAR_TRANSPORT			2.95E-03	-
PRION_DISEASES			4.39E-03	-
EPITHELIAL_CELL_SIGNALING_IN_HELICOBACTER_PYLORI_INFECTION			8.69E-03	-
TYROSINE_METABOLISM			1.09E-02	-
FATTY_ACID_METABOLISM			2.08E-02	-

Supplementary Table S4. Genes per pathway for which expression data was available

GLYCOSPHINGOLIPID_BIOSYNTHESIS_GANGLIO_SERIES

B3GALT4	GLB1	HEXB	SLC33A1	ST3GAL2	ST6GALNAC3	ST6GALNAC5	ST8SIA1	
B4GALNT1	HEXA	LCT	ST3GAL1	ST3GAL5	ST6GALNAC4	ST6GALNAC6	ST8SIA5	

GLYCOSAMINOGLYCAN_BIOSYNTHESIS_CHONDROITIN_SULFATE

B3GALT6	B3GAT3	CHPF2	CHST13	CHST3	CHSY3	UST		
B3GAT1	B4GALT7	CHST11	CHST14	CHST7	CSGALNACT1	XYLT1		
B3GAT2	CHPF	CHST12	CHST15	CHSY1	CSGALNACT2	XYLT2		

AXON_GUIDANCE

ABL1	EFNA1	EPHB1	LIMK1	NRAS	PLXNB2	RHOD	SEMA3G	SLIT2
ABLIM1	EFNA2	EPHB2	LIMK2	NRP1	PLXNB3	RND1	SEMA4A	SLIT3
ABLIM2	EFNA3	EPHB3	LRRC4C	NTN1	PLXNC1	ROBO1	SEMA4B	SRGAP1
ABLIM3	EFNA4	EPHB4	MAPK1	NTN3	PPP3CA	ROBO2	SEMA4C	SRGAP2
ARHGEF12	EFNA5	EPHB6	MAPK3	NTN4	PPP3CB	ROBO3	SEMA4D	SRGAP3
CDC42	EFNB1	FES	MET	NTNG1	PPP3CC	ROCK1	SEMA4F	UNC5A
CDK5	EFNB2	FYN	MRAS	PAK1	PPP3R1	ROCK2	SEMA4G	UNC5B
CFL1	EFNB3	GNAI1	NCK1	PAK2	PPP3R2	RRAS	SEMA5A	UNC5C
CFL2	EPHA1	GNAI2	NCK2	PAK3	PTK2	RRAS2	SEMA5B	UNC5D
CHP1	EPHA2	GNAI3	NFAT5	PAK4	RAC1	SEMA3A	SEMA6A	
CXCL12	EPHA3	GSK3B	NFATC1	PAK7	RAC2	SEMA3B	SEMA6B	
CXCR4	EPHA4	HRAS	NFATC2	PLXNA1	RAC3	SEMA3C	SEMA6C	
DCC	EPHA5	ITGB1	NFATC3	PLXNA2	RASA1	SEMA3D	SEMA6D	
DPYSL2	EPHA7	KRAS	NFATC4	PLXNA3	RGS3	SEMA3E	SEMA7A	
DPYSL5	EPHA8	L1CAM	NGEF	PLXNB1	RHOA	SEMA3F	SLIT1	

FOCAL_ADHESION

ACTB	CCND1	COMP	IBSP	ITGB8	MYL6	PIK3CD	RAP1A	TNN
ACTC1	CCND2	CRK	IGF1	JUN	MYLK	PIK3CG	RAP1B	TNR
ACTG1	CCND3	CRKL	IGF1R	KDR	MYLK2	PIK3R1	RAPGEF1	TNXB
ACTN1	CDC42	CTNNB1	ILK	LAMA1	MYLK3	PIK3R2	RELN	TTN
ACTN2	CHAD	DIAPH1	ITGA10	LAMA2	MYLK4	PIK3R3	RHOA	VASP
ACTN3	COL11A1	DOCK1	ITGA11	LAMA3	PAK1	PIK3R5	ROCK1	VAV1
ACTN4	COL11A2	EGF	ITGA2	LAMA4	PAK2	PIP5K1C	ROCK2	VAV2
AKT1	COL1A1	EGFR	ITGA2B	LAMA5	PAK3	POTEKP	SHC1	VAV3
AKT2	COL1A2	ELK1	ITGA3	LAMB1	PAK4	PPP1CA	SHC2	VCL
AKT3	COL2A1	ERBB2	ITGA4	LAMB2	PAK7	PPP1CB	SHC3	VEGFA
ARHGAP35	COL3A1	FARP2	ITGA5	LAMB3	PARVA	PPP1CC	SHC4	VEGFB
ARHGAP5	COL4A1	FIGF	ITGA6	LAMB4	PARVB	PPP1R12A	SOS1	VEGFC
BAD	COL4A2	FLNA	ITGA7	LAMC1	PARVG	PRKCA	SOS2	VTN
BCAR1	COL4A4	FLNB	ITGA8	LAMC2	PDGFB	PRKCB	SPP1	VWF
BCL2	COL4A6	FLNC	ITGA9	LAMC3	PDGFC	PRKCG	SRC	XIAP
BIRC2	COL5A1	FLT1	ITGAV	MAP2K1	PDGFD	PTEN	THBS1	ZYX
BIRC3	COL5A2	FN1	ITGB1	MAPK1	PDGFRA	PTK2	THBS2	
BRAF	COL5A3	FYN	ITGB3	MAPK10	PDGFRB	PXN	THBS3	
CAPN2	COL6A1	GRB2	ITGB4	MAPK3	PDPK1	RAC1	THBS4	
CAV1	COL6A2	GSK3B	ITGB5	MAPK8	PGF	RAC2	TLN1	
CAV2	COL6A3	HGF	ITGB6	MAPK9	PIK3CA	RAC3	TLN2	
CAV3	COL6A6	HRAS	ITGB7	MET	PIK3CB	RAF1	TNC	

ECM_RECEPTOR_INTERACTION

AGRN	COL3A1	COL6A3	GP5	ITGA3	ITGB4	LAMB1	SDC3	TNC
CD36	COL4A1	COL6A6	GP6	ITGA4	ITGB5	LAMB2	SDC4	TNN
CD44	COL4A2	DAG1	GP9	ITGA5	ITGB6	LAMB3	SPP1	TNR
CD47	COL4A4	FN1	HMMR	ITGA6	ITGB7	LAMB4	SV2A	TNXB
CHAD	COL4A6	FNDC1	HSPG2	ITGA7	ITGB8	LAMC1	SV2B	VTN
COL11A1	COL5A1	FNDC3A	IBSP	ITGA8	LAMA1	LAMC2	SV2C	VWF
COL11A2	COL5A2	FNDC4	ITGA10	ITGA9	LAMA2	LAMC3	THBS1	
COL1A1	COL5A3	FNDC5	ITGA11	ITGAV	LAMA3	RELN	THBS2	
COL1A2	COL6A1	GP1BA	ITGA2	ITGB1	LAMA4	SDC1	THBS3	
COL2A1	COL6A2	GP1BB	ITGA2B	ITGB3	LAMA5	SDC2	THBS4	

REGULATION_OF_ACTIN_CYTOSKELETON

ABI2	BAIAP2	DOCK1	FGF3	ITGA2	KRAS	PAK1	PIP4K2C	SOS1
ACTB	BCAR1	EGF	FGF4	ITGA2B	LIMK1	PAK2	PIP5K1A	SOS2
ACTC1	BDKRB1	EGFR	FGF5	ITGA3	LIMK2	PAK3	PIP5K1B	SSH1
ACTG1	BDKRB2	EZR	FGF6	ITGA4	MAP2K1	PAK4	PIP5K1C	SSH2
ACTN1	BRAF	F2	FGF7	ITGA5	MAP2K2	PAK7	POTEKP	SSH3
ACTN2	BRK1	F2R	FGF8	ITGA6	MAPK1	PDGFB	PPP1CA	TIAM1
ACTN3	CD14	FGD1	FGF9	ITGA7	MAPK3	PDGFRA	PPP1CB	TIAM2
ACTN4	CDC42	FGD3	FGFR1	ITGA8	MATK	PDGFRB	PPP1CC	TMSB4X
APC	CFL1	FGF1	FGFR2	ITGA9	MOS	PFN1	PPP1R12A	TMSB4XP8
APC2	CFL2	FGF10	FGFR3	ITGAD	MRAS	PFN2	PPP1R12B	TMSB4Y
ARHGAP35	CHRM1	FGF11	FGFR4	ITGAE	MSN	PFN3	PTK2	TTN
ARHGEF1	CHRM2	FGF12	FN1	ITGAL	MYH10	PFN4	PXN	VAV1
ARHGEF12	CHRM3	FGF13	GIT1	ITGAM	MYH14	PIK3CA	RAC1	VAV2
ARHGEF4	CHRM4	FGF14	GNA12	ITGAV	MYH9	PIK3CB	RAC2	VAV3
ARHGEF6	CHRM5	FGF16	GNA13	ITGAX	MYL1	PIK3CD	RAC3	VCL
ARHGEF7	CRK	FGF17	GNG12	ITGB1	MYL3	PIK3CG	RAF1	WAS
ARPC1A	CRKL	FGF18	GSN	ITGB2	MYLK	PIK3R1	RDX	WASF1
ARPC1B	CSK	FGF19	HRAS	ITGB3	MYLK2	PIK3R2	RHOA	WASF2
ARPC2	CYFIP1	FGF2	IQGAP1	ITGB4	MYLK3	PIK3R3	ROCK1	WASL
ARPC3	CYFIP2	FGF20	IQGAP2	ITGB5	MYLK4	PIK3R5	ROCK2	
ARPC4	DIAPH1	FGF21	IQGAP3	ITGB6	NCKAP1	PIKFYVE	RRAS	
ARPC5	DIAPH2	FGF22	ITGA10	ITGB7	NCKAP1L	PIP4K2A	RRAS2	
ARPC5L	DIAPH3	FGF23	ITGA11	ITGB8	NRAS	PIP4K2B	SLC9A1	

HEDGEHOG_SIGNALING_PATHWAY

BMP2	BTRC	CSNK1G3	GSK3B	PRKX	STK36	WNT2	WNT6	ZIC2
BMP4	CSNK1A1	DHH	HHIP	PRKY	SUFU	WNT2B	WNT7A	
BMP5	CSNK1A1L	FBXW11	IHH	PTCH1	WNT1	WNT3	WNT7B	
BMP6	CSNK1D	GAS1	LRP2	PTCH2	WNT10A	WNT3A	WNT8A	
BMP7	CSNK1E	GLI1	PRKACA	RAB23	WNT10B	WNT4	WNT8B	
BMP8A	CSNK1G1	GLI2	PRKACB	SHH	WNT11	WNT5A	WNT9A	
BMP8B	CSNK1G2	GLI3	PRKACG	SMO	WNT16	WNT5B	WNT9B	

WNT_SIGNALING_PATHWAY

APC	CSNK1A1L	DVL2	GSK3B	NKD2	PPP3CC	RBX1	TBL1XR1	WNT3A
APC2	CSNK1E	DVL3	JUN	NLK	PPP3R1	RHOA	TBL1Y	WNT4
AXIN1	CSNK2A1	EP300	LEF1	PLCB1	PPP3R2	ROCK1	TCF7	WNT5A
AXIN2	CSNK2A2	FBXW11	LRP5	PLCB2	PRICKLE1	ROCK2	TCF7L1	WNT5B
BTRC	CSNK2B	FOSL1	LRP6	PLCB3	PRICKLE2	RUVBL1	TCF7L2	WNT6
CACYBP	CTBP1	FRAT1	MAP3K7	PLCB4	PRKACA	SENP2	TP53	WNT7A
CAMK2A	CTBP2	FRAT2	MAPK10	PORCN	PRKACB	SFRP1	VANGL1	WNT7B
CAMK2B	CTNNB1	FZD1	MAPK8	PPARD	PRKACG	SFRP2	VANGL2	WNT8A
CAMK2D	CTNNBIP1	FZD10	MAPK9	PPP2CA	PRKCA	SFRP4	WIF1	WNT8B
CAMK2G	CUL1	FZD2	MMP7	PPP2CB	PRKCB	SFRP5	WNT1	WNT9A
CCND1	CXXC4	FZD3	MYC	PPP2R1A	PRKCG	SIAH1	WNT10A	WNT9B
CCND2	DAAM1	FZD4	NFAT5	PPP2R1B	PRKX	SKP1	WNT10B	
CCND3	DAAM2	FZD5	NFATC1	PPP2R2A	PRKY	SMAD2	WNT11	
CER1	DKK1	FZD6	NFATC2	PPP2R2B	PSEN1	SMAD3	WNT16	
CHP1	DKK2	FZD7	NFATC3	PPP2R2C	RAC1	SMAD4	WNT2	
CREBBP	DKK4	FZD8	NFATC4	PPP3CA	RAC2	SOX17	WNT2B	
CSNK1A1	DVL1	FZD9	NKD1	PPP3CB	RAC3	TBL1X	WNT3	

TGF_BETA_SIGNALING_PATHWAY

ACVR1	BMP5	COMP	GDF7	LEFTY1	PPP2CB	SMAD1	SP1	THBS4
ACVR1B	BMP6	CREBBP	ID1	LEFTY2	RBL1	SMAD2	TFDP1	TNF
ACVR1C	BMP7	CUL1	ID2	LTBP1	RBL2	SMAD3	TGFB1	ZFYVE16
ACVR2A	BMP8A	DCN	ID3	MAPK1	RBX1	SMAD4	TGFB2	ZFYVE9
ACVR2B	BMP8B	E2F4	ID4	MAPK3	RHOA	SMAD5	TGFB3	
ACVRL1	BMPR1A	E2F5	IFNG	MYC	ROCK1	SMAD6	TGFBR1	
AMH	BMPR1B	EP300	INHBA	NODAL	ROCK2	SMAD7	TGFBR2	
AMHR2	BMPR2	FST	INHBB	NOG	RPS6KB1	SMAD9	THBS1	
BMP2	CDKN2B	GDF5	INHBC	PITX2	RPS6KB2	SMURF1	THBS2	
BMP4	CHRD	GDF6	INHBE	PPP2CA	SKP1	SMURF2	THBS3	

Supplementary Table S5. List of genes that were predicted as direct targets by the combined use of 3 independent target prediction algorithms.

miRNA	Gene Symbol	Gene Name	Spearman's rho	P value
miR-30b	*ABL1*	ABL Proto-Oncogene 1, Non-Receptor Tyrosine Kinase	-0.032	0.63
	CFL2	Cofilin 2	0.107	0.11
	DPYSL2	Dihydropyrimidinase Like 2	0.072	0.28
	EFNA3	Ephrin A3	0.121	0.07
	GNAI2	G Protein Subunit Alpha I2	-0.030	0.65
	NFAT5	Nuclear Factor Of Activated T-Cells 5	-0.286	<0.0001
	NFATC2	Nuclear Factor Of Activated T-Cells 2	-0.009	0.90
	NFATC3	Nuclear Factor Of Activated T-Cells 3	0.067	0.32
	PLXNA2	Plexin A2	-0.077	0.25
	PLXNC1	Plexin C1	-0.007	0.92
	PPP3R1	Protein Phosphatase 3 Regulatory Subunit B, Alpha	-0.331	<0.001
	RASA1	RAS P21 Protein Activator 1	-0.083	0.22
	SEMA6B	Semaphorin 6B	-0.198	0.003
	SEMA6D	Semaphorin 6D	0.178	0.007
	SRGAP3	SLIT-ROBO Rho GTPase Activating Protein 3	-0.018	0.79
	UNC5C	Unc-5 Netrin Receptor C	0.295	<0.0001
Let-7i	*ACVR1C*	Activin A Receptor Type 1C	-0.113	0.09
	CHRD	Chordin	0.422	<0.0001
	COL1A2	Collagen Type I Alpha 2 Chain	0.561	<0.0001
	COL3A1	Collagen Type III Alpha 1 Chain	0.576	<0.0001
	COL4A6	Collagen Type IV Alpha 6 Chain	-0.258	0.0001
	COL5A2	Collagen Type V Alpha 2 Chain	0.512	<0.0001
	E2F5	E2F Transcription Factor 5	0.076	0.25
	FNDC3A	Fibronectin Type III Domain Containing 3A	-0.295	<0.0001
	GDF6	Growth Differentiation Factor 6	0.053	0.54
	ITGB3	Integrin Subunit Beta 3	0.196	0.0031
	TGFBR1	Transforming Growth Factor Beta Receptor 1	0.346	<0.0001

Supplementary Table S6. Gene assays used to measure mRNA expression of the six MiRNAs and 3 reference genes

INDEX	Gene Symbol	Gene Name	qPCR detection method	Assay ID ThermoFisher Scientific
Candidate miR	***MIR320***	microRNA 320	TaqMan® MicroRNA Assay	384
Candidate miR	***MIR221***	microRNA 221	TaqMan® MicroRNA Assay	2277
Candidate miR	***MIR30B***	microRNA 30b	TaqMan® MicroRNA Assay	2218
Candidate miR	***MIR10B***	microRNA 10b	TaqMan® MicroRNA Assay	524
Candidate miR	***MIR885***	microRNA 885	TaqMan® MicroRNA Assay	602
Candidate miR	***MIRLET7I***	microRNA let-7i	TaqMan® MicroRNA Assay	2296
Reference miR	***MIR16-1***	microRNA 16-1	TaqMan® MicroRNA Assay	391
Reference miR	***RNU6B***	RNA, U6 small nuclear 6, pseudogene	TaqMan® MicroRNA Assay	1093
Reference miR	***SNORD44***	small nucleolar RNA, C/D box 44	TaqMan® MicroRNA Assay	1094

Supplementary Fig. S1. Supplemental Fig. S1 displays the correlation between the miRNAs (scatter plots) with their respective spearman's correlation coefficient and p values. The distribution of the expression levels of the six miRNAs are displayed on the diagonal with their respective p values (Shapiro-Wilk test).

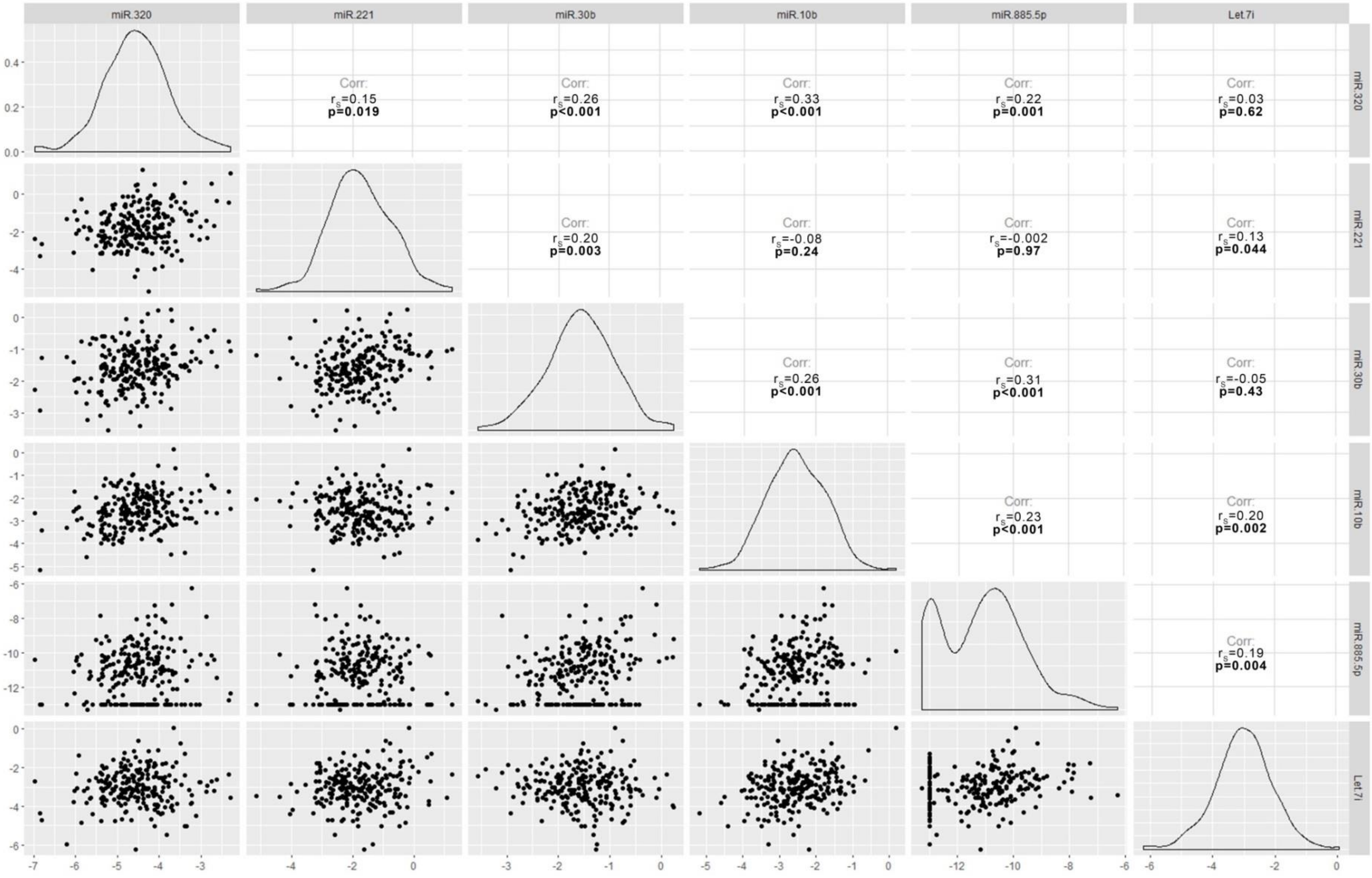

Supplement: Supplementary file 1 — Supplementary information file [file 41598_2018_22532_MOESM1_ESM.pdf]
